# On the relationship between energy input to the ionosphere and the ion outflow flux under different solar zenith angles

**DOI:** 10.1186/s40623-021-01532-y

**Published:** 2021-11-06

**Authors:** Naritoshi Kitamura, Kanako Seki, Kunihiro Keika, Yukitoshi Nishimura, Tomoaki Hori, Masafumi Hirahara, Eric J. Lund, Lynn M. Kistler, Robert J. Strangeway

**Affiliations:** 1 Department of Earth and Planetary Science, Graduate School of Science, The University of Tokyo, Tokyo, Japan; 2 Department of Electrical and Computer Engineering and Center for Space Physics, Boston University, Boston, MA USA; 3 Institute for Space-Earth Environmental Research, Nagoya University, Furo-cho, Chikusa-ku, Nagoya, Japan; 4 Institute for the Study of Earth, Oceans, and Space, University of New Hampshire, Durham, NH USA; 5 College Brook Scientific, Durham, NH USA; 6 Department of Physics, University of New Hampshire, Durham, NH USA; 7 Department of Earth, Planetary, and Space Sciences, University of California, Los Angeles, CA USA

**Keywords:** Auroral ion outflow, Polar ionosphere, Auroral precipitation, FAST satellite, Cleft ion fountain, Ion conics, Ion beams

## Abstract

The ionosphere is one of the important sources for magnetospheric plasma, particularly for heavy ions with low charge states. We investigate the effect of solar illumination on the number flux of ion outflow using data obtained by the Fast Auroral SnapshoT (FAST) satellite at 3000–4150 km altitude from 7 January 1998 to 5 February 1999. We derive empirical formulas between energy inputs and outflowing ion number fluxes for various solar zenith angle ranges. We found that the outflowing ion number flux under sunlit conditions increases more steeply with increasing electron density in the loss cone or with increasing precipitating electron density (> 50 eV), compared to the ion flux under dark conditions. Under ionospheric dark conditions, weak electron precipitation can drive ion outflow with small averaged fluxes (~ 10^7^ cm^−2^ s^−1^). The slopes of relations between the Poynting fluxes and outflowing ion number fluxes show no clear dependence on the solar zenith angle. Intense ion outflow events (> 10^8^ cm^−2^ s^−1^) occur mostly under sunlit conditions (solar zenith angle < 90°). Thus, it is presumably difficult to drive intense ion outflows under dark conditions, because of a lack of the solar illumination (low ionospheric density and/or small scale height owing to low plasma temperature).

**Graphical abstract:**

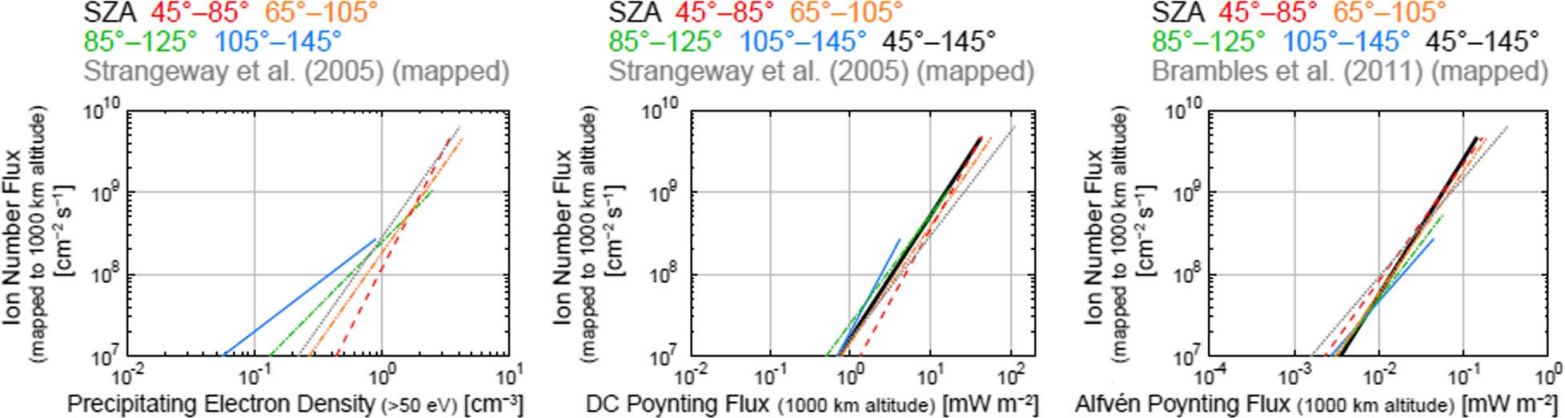

## Main text

### Introduction

Quantifying the properties of outflowing ionospheric ions is one of the most important subjects for magnetospheric studies, because the physical characteristics of the magnetosphere are modulated significantly by outflowing ions. Many satellite observations have demonstrated that ionospheric O^+^ ions are supplied to the plasma sheet and inner magnetosphere, especially during geomagnetically active periods (e.g., Daglis 1997; Yao et al. 2008a; Ebihara et al. 2009; Mouikis et al. 2010, 2019; Ohtani et al. 2011; Kronberg et al. 2012, 2015; Maggiolo and Kistler 2014; Kistler and Mouikis 2016; Keika et al. 2018a, 2018b; Mitani et al., 2019; Kistler et al. 2019). Various modeling and observational studies have suggested that an increase in the ionospheric O^+^ ions in the magnetosphere would affect reconnection processes (e.g., Shay and Swisdak 2004; Karimabadi et al. 2011; Liu et al. 2015; Fuselier et al., 2019; Tenfjord et al. 2019), location of the tail reconnection (Brambles et al. 2010; Garcia et al. 2010; Wiltberger et al. 2010; Yu and Ridley 2013), growth and propagation of electromagnetic ion cyclotron waves (e.g., Omidi et al. 2013; Denton et al. 2014; Nosé et al., 2020), and development and decay of the ring current (e.g., Hamilton et al. 1988; Keika et al. 2006; Glocer et al. 2009a,b, 2013; Welling et al. 2011; Ilie et al. 2015; Menz et al. 2019). Moreover, modeling studies by Brambles et al. (2011, 2013), Ouellette et al. (2013), Varney et al. (2016), and Zhang et al. (2020) showed that inclusion of O^+^ ion outflows can change the mode of global magnetospheric convection: from steady convection to sawtooth oscillations. Observations and effects of O^+^ ions in the magnetosphere are summarized in more detail in review papers by Keika et al. (2013), Kronberg et al. (2014), Welling et al. (2016a), and Yamauchi (2019).

Some of the O^+^ ions that have reached the magnetosphere are transported to the boundary regions or the distant tail by their drift motion and eventually lost to the interplanetary space. Past studies reported that they can escape through the boundary layer (Zong et al. 2004; Bouhram et al. 2005; Cohen et al. 2016; Zeng et al. 2020), plasma mantle (Slapak et al. 2017; Schillings et al. 2019, 2020), and/or distant tail (Seki et al. 1998; Kistler et al. 2010). Additionally, some O^+^ ions are lost as energetic neutral atoms due to the charge exchange process (Keika et al. 2006; Valek et al. 2018). Thus, the understanding of ion outflow from the ionosphere also contributes to the understanding of atmospheric loss from magnetized planets.

To include ion outflows from the ionosphere in global magnetospheric simulations, moments of the outflowing ion distribution function can be used as the boundary conditions at the inner boundary, which is typically located at ~ 2.5 Earth radii (*R*_E_) in geocentric distance. If temporal variations of the ion outflows are important for the studies, time-dependent inner boundary conditions are necessary. There have been two approaches for it: one is to use ion outflows from ionospheric simulations (e.g., Schunk and Sojka 1997; Barakat and Schunk 2006; Glocer et al. 2012, 2018, 2020; Pham et al. 2021; Varney et al. 2015, 2016; Welling et al. 2015, 2016b), and the other is to use empirical relations between energy inputs and ion outflow fluxes (Fok et al. 2006, 2011; Moore et al. 2007, 2010; Brambles et al. 2010, 2011, 2013; Damiano et al. 2010; Peroomian et al. 2011; Ouellette et al. 2013). The present study provides such empirical relations that include effects of the solar illumination. Such empirical relations may also be useful for rough validation of ion outflow simulations.

Using data obtained by the Fast Auroral SnapshoT (FAST) satellite, statistical studies by Strangeway et al. (2005) and Brambles et al. (2011) indicated that fluxes of ion outflows are correlated well with the precipitating electron density (> 50 eV), electron density in the loss cone (> 50 eV), and DC and Alfvén Poynting fluxes. The soft electron precipitation is expected to contribute to electron heating, which cause an increase in electron scale height due to enhancements of the ambipolar electric field at the topside ionosphere, while the Poynting flux is expected to contribute to Joule dissipation at the ionosphere which cause an increase in ion scale height (Strangeway et al. 2005). DC and Alfvén Poynting fluxes correspond to quasistatic electromagnetic energy input and earthward-flowing electromagnetic energy flux at ultralow frequencies, respectively. Further detailed explanations about the DC and Alfvén Poynting fluxes are described in the Supplemental Material of Brambles et al. (2011). Strangeway et al. (2005) and Brambles et al. (2011) derived empirical formulas between these energy inputs to the ionosphere and outflowing ion number fluxes at ~ 4000 km altitude using the data obtained near the cusp region in the dayside (mostly in the postnoon sector) during and before/after a geomagnetic storm (24–26 September 1998, which included ~ 30 orbit passes). Zheng et al. (2005) also derived similar empirical formulas using data obtained by the Polar spacecraft at ~ 5000 km altitude (37 events during year 2000, mostly in the dayside, not focused on a specific geomagnetic storm). Recently, Zhao et al. (2020) updated the empirical formulas derived by Strangeway et al. (2005) and Brambles et al. (2011) using the mass resolved ion data derived by the FAST satellite during the same geomagnetic storm as previously studied by them. Hatch et al. (2020a) focused on the east–west magnetic field fluctuations, and investigated the relation between the magnetic field fluctuations and ion outflows around the cusp for four geomagnetic storms in various seasons. They examined the correlation of the ion outflow flux with magnetic fluctuations and showed that the outflow flux has a smaller increase rate with increasing amplitude of the east–west fluctuations in winter than in summer and equinox seasons.

The solar illumination (or season) strongly affects the ionosphere in terms of the condition under which the ionosphere receives energy inputs from the magnetosphere (Garner et al. 2010; Hatch et al. 2020b; Zhang et al. 2010). A statistical study by Kitamura et al. (2011) that used data from the Akebono and Intercosmos satellites, and the European incoherent scatter Svalbard radar reported that the temperature and scale height of the background thermal plasma in the topside ionosphere are strongly controlled by the solar zenith angle (SZA), which is the angle between the sun direction and the local zenith direction at the ionospheric footprint of the satellite. It causes large seasonal dependence of the electron density (high density in the summer season (~ small SZA)) around 2000 km altitude in the polar region under quiet geomagnetic conditions (Kitamura et al. 2009). Using data obtained by the Defense Meteorological Satellite Program, Ma et al. (2018) investigated the effect of solar illumination on ion upflows and found that the effect is not simple. For example, they showed that high-speed upflow can occur under dark conditions, while upflows with large densities can occur under sunlit conditions. Some statistical studies using incoherent scatter radar data have identified seasonal variation of the occurrence frequency of ion upflows (Foster et al. 1998; Liu et al. 2001; Buchert et al. 2004; Ji et al. 2019; Ren et al. 2020), although the seasonal variation seems to depend on the observed altitude and/or location of the radar. The occurrence of upward ion beams is also strongly affected by the solar illumination; the occurrence rate is lower under sunlit ionospheric conditions (on the basis of measurements below ~ 4000 km altitude) (Cattell et al. 2013), and is also lower in winter, which mostly corresponds to dark conditions (on the basis of measurements around ~ 6000 km altitude) (Collin et al. 1998). The occurrence frequency of ion conics (or transversely accelerated ions) at ~ 1500 km altitude is higher in winter, which corresponds mostly to dark conditions (Klumpar 1979; Norqvist et al. 1998). Broadband extremely low-frequency waves (observed below ~ 10,000 km altitude (Kasahara et al. 2001)) and electromagnetic ion cyclotron waves (500–4000 km (Saito et al. 1987) and ~ 1500 km altitude (Erlandson and Zanneti 1998; Hamrin et al. 2002)), which are thought to be the main driving processes of ion conics, also tend to be preferentially generated under winter and/or dark conditions. These various types of observations support the importance of solar illumination (ionospheric conditions) for ion outflows. Thus, the solar illumination may affect the empirical relationships between the energy inputs and outflowing ion number fluxes. Since the SZA at the magnetic footprint of the events used by Strangeway et al. (2005) and Brambles et al. (2011) was smaller than 92°, their empirical formulas represent those under sunlit ionospheric conditions.

Some modeling studies of ion outflows have shown a seasonal dependence (Demars and Schunk 2001, 2002) or interhemispheric asymmetry (Barakat et al. 2015; Glocer et al. 2020) of ion outflows, although the models are incomplete because physical processes of ion outflows have not been fully understood yet.

To understand how strongly (sunlit or dark) ionospheric conditions affect ion outflows, we derive empirical formulas of outflowing ion number fluxes as a function of each energy input (electron density in the loss cone (> 50 eV), precipitating electron density (> 50 eV), DC and Alfvén Poynting fluxes) for a wide SZA range (45°–145°), using data obtained by the FAST satellite (3000–4150 km altitude). The structure of this manuscript goes in the following way: “Dataset and selection of ion outflow events” section describes the datasets and the event selection criteria we used. “SZA dependence of ion number fluxes” and “SZA dependence of the empirical relation between energy inputs and the ion number flux” sections present the results of our data analysis, followed by some discussions in “Discussion” section.

## Dataset and selection of ion outflow events

The FAST satellite was launched in 1996 with an initial perigee, apogee, and inclination of 350 km, 4175 km, and 83°, respectively. The satellite was spin-stabilized with a spin period of ~ 5 s. The spin axis was nearly normal to the orbital plane (Carlson et al. 1998). We used data obtained in four intervals between 7 January 1998 and 5 February 1999 (7 January 1998–4 April 1998 (North), 3 May 1998–20 July 1998 (South), 31 July 1998–26 October 1998 (North), and 15 December 1998–6 February 1999 (South)). These periods are suitable for studying the impact of SZA, because the orbital plane of the FAST satellite tended to be aligned to the noon–midnight meridian when the apogee stayed near the pole. This orbit configuration enables the satellite to traverse the auroral zone (or cusp) at various SZAs even in a single day, owing to the shift of the magnetic pole from the rotational axis. In contrast, the satellite can only measure very limited specific SZA repeatedly in cases where the orbital plane was closely aligned to the dawn–dusk meridian. The monthly mean F10.7 index ranged between 93.4 and 150.1, which is almost the same level as the solar maximum of Solar cycle 24.

The invariant latitude (ILAT) and magnetic local time (MLT) of the satellite were provided as orbital information for the FAST satellite. In the orbital information data, an offset tilted dipole (dipole geographic position: (− 402.199, 287.504, 195.908) km, dipole orientation: latitude 79.3637°, longitude 288.454°) was used. ILAT is defined as the latitude of the dipole magnetic field line where the satellite was located, at 6371.2 km from the center of the dipole. MLT is defined as the local time of the dipole magnetic field line discussed above.

The electron and ion (without mass separation) spectrometers (EESA and IESA) measured two-dimensional (360°) electron and ion velocity distributions with an angular resolution of 11.25° (32 bins) or 5.625° (64 bins in limited periods of IESA) in an energy range of ~ 4 eV–32 keV and ~ 3 eV–24 keV, respectively (Carlson et al. 2001). During the data periods for the present study, the EESA and IESA covered the energy ranges with 48 energy steps. The electron density in the loss cone (see “Empirical relations between the electron density in the loss cone and the ion number flux” section for the definition) and the precipitating electron density, which was proposed by Strangeway et al. (2005) and was calculated using the energy flux and the number flux (see “Empirical relations between the precipitatingelectron density and the ion number flux” section for the definition), include electrons in the energy range of 50 eV–32 keV. This low energy limit (50 eV) is set to avoid the contamination of ionospheric photoelectrons, following Strangeway et al. (2005). Background counts were subtracted from the IESA data using count rates in the loss cone in the upward direction (Appendix A1).

The low energy limit for calculations of field-aligned (upward positive) ion number fluxes was set to 10 eV to reduce the influence of small changes in the spacecraft potential and the effect of spacecraft motion (ram effect) on the calculation of ion number flux. In cases where the orbital velocity of FAST was not perpendicular to the magnetic field, sometimes artificial fluxes owing to the ram effect became significant below ~ 10 eV; the spacecraft velocity of ~ 6.2 km s^−1^ (~ 3000 km altitude) corresponds to the energy of ~ 3.2 eV for O^+^ ions. Note that this lower limit (10 eV) is higher than that used by Strangeway et al. (2005) (4 eV). This change is done to find a much larger number of events quantitatively (not with visual inspection) from times when the apogee is at various latitudes.

**Fig. 1:**
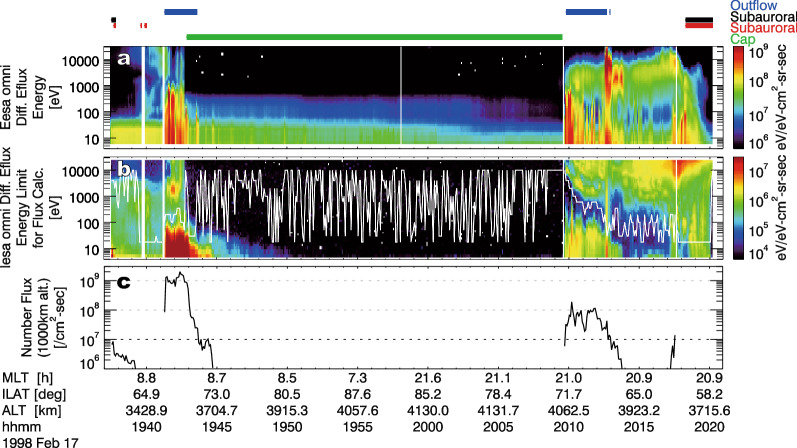
Example of observations at high latitudes. Omnidirectional energy–time spectrograms of differential energy flux of **a** electrons and **b** ions, and **c** number fluxes of ions observed by IESA. Blue and green bars indicate the periods of the outflow regions (“Dataset and selection of ion outflow events” section) and the polar cap (Appendix A4), respectively. Black and red bars indicate the periods that are selected for the identification of the subauroral zone, and they are related to the high background count rates and double loss cones, respectively (Appendix A5). A white polygonal line in Fig. 1b is the upper energy limit for the calculation of number flux of ions

At magnetic footprints of the cusp, the boundary layer, and the plasma sheet, high-energy ions from the magnetosphere or the solar wind precipitate into the ionosphere. Since these populations contribute negatively to the ion number flux, such contribution must be separated from that of the outflowing ions. In the present study, the contribution of the precipitating ions was separated by referring to their energy difference: the energy of outflowing ions is lower than that of the precipitating ions. As described above, the lower energy limit of the calculation of the field-aligned (upward positive) ion number flux (IESA) was fixed to 10 eV. As the upper energy limit, about 18, 30, 50, 100, 200, 350, 600, 1000, 2000, 4000, and 10,000 eV (per 3 or 4 energy bin, except for 4–10 keV that includes 5 energy bins) were used to calculate field-aligned ion number fluxes. In each 5-s interval, the largest field-aligned ion number flux was used as the number flux of outflowing ions, and the upper energy limit for the largest ion number flux was also recorded as the boundary between the outflowing and precipitating components. Figure 1 shows an example of the data during the main phase of a geomagnetic storm (*Kp* = 4, *AL* =  − 500– − 1000 nT). Electrons and high-energy (above the white line) ions from the cusp/cleft and plasma sheet were detected at ~ 19:42 UT and after ~ 20:10 UT, respectively. A white polygonal line in Fig. 1b is the upper energy limit of outflowing ions selected as described above. In the region where the outflowing low-energy ion number flux (Fig. 1c) is large (> 10^7^ cm^−2^ s^−1^), the precipitating ions with high energies are appropriately separated from the outflowing low-energy component. The magnetic field (Elphic et al. 2001) and electric field (Ergun et al. 2001) data were used to derive Poynting fluxes. The Poynting flux was calculated using the electric field almost along the orbital velocity vector (E_along_V_) of the satellite (1 s average), which was measured by the probes in the spin plane, and the deviation from the International Geomagnetic Reference Field 11th generation (IGRF-11) model magnetic field (Finlay et al. 2010) perpendicular to the orbital velocity vector (δB_perp_V_) (1 s average). For calculations of Poynting fluxes, we calculated a simple moving average for each data point (E_along_V_ and δB_perp_V_) using a 7-point window (= 7 s), which corresponds to the spatial resolution of ~ 40 km at the altitude of the satellite. The Poynting flux of DC fields (DC Poynting flux: < 0.125 Hz) was calculated as the vector product of the moving averaged values of E_along_V_ and δB_perp_V_ (Strangeway et al. 2005). On the other hand, the Poynting flux of Alfvénic waves (Alfvén Poynting flux: 0.125–0.5 Hz) was the vector product of residuals of E_along_V_ and δB_perp_V_ after subtraction of the running averaged values (Brambles et al. 2011). For these Poynting fluxes, a positive value corresponds to a downward Poynting flux. Note that the electric field perpendicular to the velocity vector of the satellite is not derived owing to lack of reliable measurements of the electric field along the spin axis. Thus, the magnitude of the Poynting fluxes is underestimated, and this incomplete Poynting flux measurement probably contributes to somewhat large scatter in some results of the present analysis on the relationship between the Poynting fluxes and the ion flux (“Empirical relations between the DC poynting flux and the ion number flux” and “Empirical relations between the Alfvén poynting flux and the ion number flux” sections).

Ion number fluxes, electron energy and number fluxes, and Poynting fluxes were mapped to 1000 km altitude, assuming the dipole magnetic field: the fluxes were multiplied by the ratio of the dipole magnetic field strength at 1000 km altitude and that at the altitude of the satellite. The particle, magnetic field, and Poynting flux data were averaged over 5-s intervals (~ 1 spin) after removing erroneous data (Appendix A2), resulting in the dataset with a uniform time resolution of 5 s.

Before identification of ion outflow events, intervals of significant negative spacecraft charging, which causes artificially large ion number fluxes, were identified, and were treated as data gaps. Although such negative spacecraft charging was rare around the apogee, even a small number of such events can affect the present statistical study, because real events with very large ion number flux were also rare. A more detailed explanation about the intervals is described in Appendix A3.

We focused on full auroral zone (or cusp) crossings as much as possible. Thus, the data obtained during orbit passes that included observations of the polar cap longer than 200 s (40 data points) were used for the present statistical study. The polar cap was defined with the threshold of a mean differential energy flux (< 10^4.6^ eV cm^−2^ s^−1^ sr^−1^ eV^−1^) of the 5-s averaged low-energy ion data (110 eV–24 keV). The threshold of differential energy flux is identical to that used by Andersson et al. (2004). A more detailed explanation about the selection of the polar cap is described in Appendix A4. A green bar above the top of Fig. 1 is an example of the identified polar cap periods. The orbit passes at high latitudes (ILAT > 45°) was divided into an inbound and an outbound part.

As the candidates of the outflow region, continuous (≥ 10 s, ≥ 2 data points) data points with mapped ion number flux larger than 10^7^ cm^−2^ s^−1^ were selected. Blue bars at the top of Fig. 1 are an example of the candidates. This threshold flux was determined from inspection of the data. Although contamination owing to solar radiation affects the flux in some cases (Appendix A4), the effect was small at least if the real flux was larger than this threshold flux. To focus on ion outflows in the auroral zone and cusp, candidates in the subauroral zone or lower latitude, which were rare, were excluded. Details of this identification are described in Appendix A5.

Because the dataset (7 January 1998–5 February 1999) is very large, there are some candidates of outflow regions that are not appropriate to use. Data from the inbound or outbound part were not used for the statistical analysis if any of the outflow regions met at least one of the following criteria:

1. The total time length of the outflow region was < 20 s (4 data points).
2. Edges of the outflow region were located ≤ 1° in ILAT from the low and high latitude limit of the data.
3. A total of data gap periods of IESA or EESA around (≤ 1° in ILAT from the edge) the outflow region exceeded 25% of the total time length of the data obtained in the outflow region.
4. Errors of the magnetic field data occurred (Appendix A2-1).
5. Any of the data points of the selected outflow region were obtained at < 3000 km altitude.

This last criterion is set to limit the sampled range of altitude for the selected events and to avoid negative charging and high spacecraft velocity. Because the plasma density increases exponentially with decreasing altitude (Kitamura et al. 2009, 2011), the ion flux due to the ram effect increases drastically, and the ram effect creates an apparent flux increase at ~ 10 eV at low altitudes. This criterion also helps to reduce altitude dependence of the outflowing ion number fluxes above 10 eV. Although the field-aligned ion number fluxes are expected to be almost continuous in the direction of altitude on average at ~ 3000 km altitude where the local production is negligible, ions must have been energized to > 10 eV at somewhere below the altitude of the satellite to exceed the lower energy limit (10 eV) of the present analysis. At low altitudes, outflowing ions below 10 eV may be dominant, and the outflowing ion number fluxes may be significantly underestimated due to the lower energy limit, if similar data obtained at very low altitudes. The limitation due to the lower energy limit of 10 eV is also discussed in “Discussion” section.

We averaged ion number fluxes from IESA, electron densities in the loss cone, and Poynting fluxes of DC fields and Alfvénic waves during all candidates of the outflow region together in each inbound or outbound pass using the latitudinal width in ILAT in each 5-s data as the weight. By using this weight for the averaging, we can treat the data as if the satellite had crossed the auroral zone in the latitudinal direction with a constant velocity, regardless of its orbit, which usually crosses the auroral zone obliquely. The averaged data are counted as 1 event. The averaged SZA in each of these outflow events was calculated. We used the product of the latitudinal width in ILAT (Δ_ILAT_5s_) and the mapped ion number flux (*F*_i_1000_5s_) as the weight (*w* = *F*_i_1000_5s_Δ_ILAT_5s_) for each data point, when we calculate the arithmetic mean of the SZA values at the center of the data points in each outflow event (Σ(SZA*w*)/Σ*w*). Because this weight is proportional to the contribution of each 5-s data to the averaged ion number flux, the SZA at the data points that contributed heavily to the averaged ion number flux also contributes heavily to the averaged SZA. In total, we find 1569 events, which provide the starting dataset for this study. Poynting fluxes were available in 1448 events out of the total 1569 events (Appendix A2-2).

## SZA dependence of ion number fluxes

**Fig. 2:**
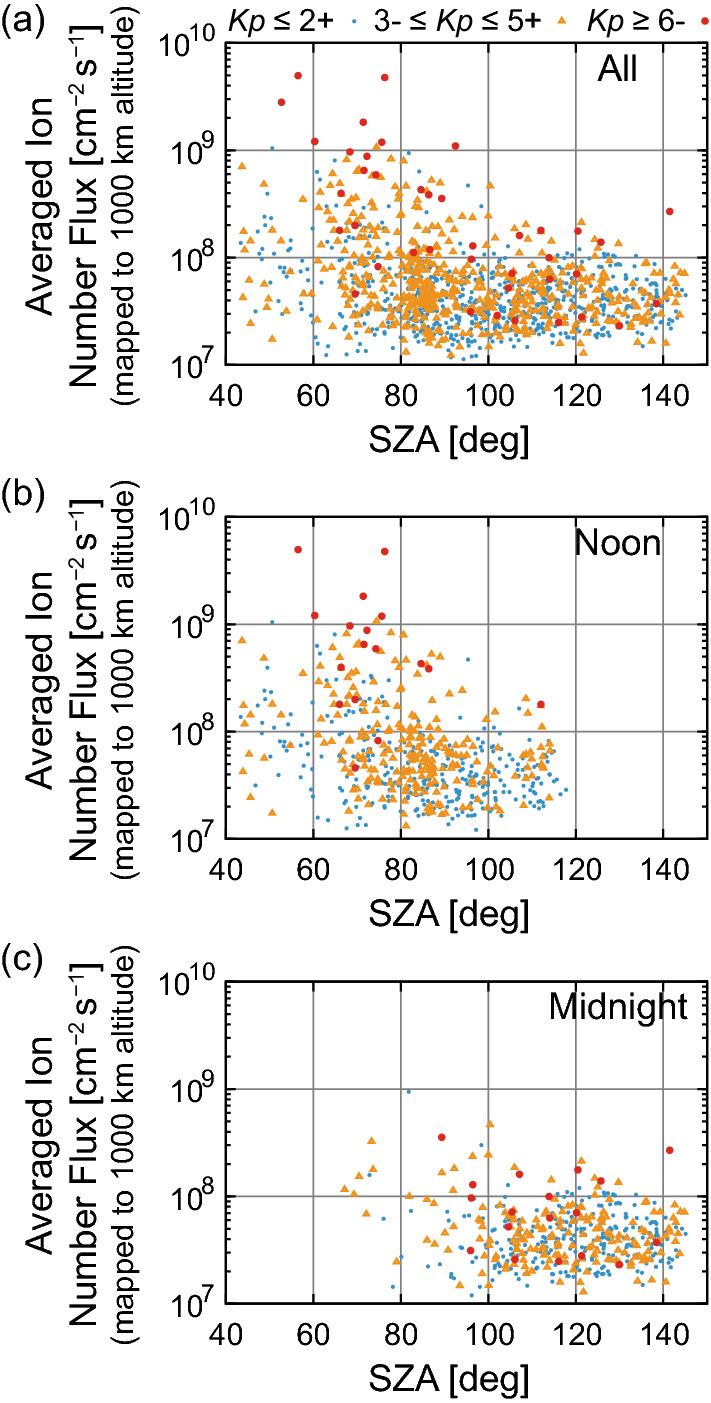
SZA distributions of averaged ion number flux in each event. Events at **a** all MLT, **b** only around noon (0800–1600 MLT), and **c** only around midnight (2200–2400 or 0000–0400 MLT). Different symbols and colors indicate different levels of the *Kp* index

Figure 2a indicates the outflowing ion number flux (mapped to 1000 km altitude) for various SZA values (1569 events). Outflow events with large averaged fluxes (> 10^8^ cm^−2^ s^−1^) occurred mostly under sunlit ionospheric conditions (SZA < 90°), although events during high geomagnetic activity (large *Kp* index) occurred also under dark conditions. This result indicates that the effect of the solar illumination (likely high ionospheric density and/or large scale height owing to high plasma temperature (Kitamura et al. 2011)) is important for the occurrence of ion outflows with large number fluxes. This result is consistent with the seasonal dependence of ion outflow discussed by Yau et al. (1985), which indicates that more O^+^ outflow occurs in summer than winter. Note that the outflowing ion flux in Fig. 2a cannot simply be considered as averaged fluxes for specific *Kp* levels, since events of small (< 10^7^ cm^−2^ s^−1^) ion number fluxes are not included in the statistics due to the threshold.

Only the events near noon (0800–1600 MLT) are plotted in Fig. 2b. The events that include any data point (before averaging) outside of the 0800–1600 MLT range are not plotted. The figure shows that most of the events with large ion number fluxes occurred near noon. In contrast, the lack of ion outflow events with large number fluxes near midnight (2000–2400 or 0000–0400 MLT) (Fig. 2c) is consistent with the importance of solar illumination for the occurrence of ion outflows with large number fluxes. Because the auroral zone around midnight is rarely illuminated by the sun, presumably it is difficult to drive ion outflow with large number fluxes around midnight. As described in “Dataset and selection of ion outflow events” section, the orbital plane of the FAST satellite tended to be aligned to the noon–midnight meridian when the apogee stayed near the pole. Thus, auroral oval crossings are concentrated around noon and midnight. Because the overlap (around SZA of 100°) of events around noon and midnight is limited, it is difficult to investigate the difference in the empirical relation around noon and that around midnight at the same SZA. Detailed analysis of the MLT effect is beyond the scope of the present study.

Schillings et al. (2017, 2018) investigated O^+^ ion outflows during large geomagnetic storms using data obtained by the Cluster spacecraft as extreme cases, and reported large magnitudes of O^+^ number flux (event mean) between 3.5 × 10^7^ and 2 × 10^9^ cm^−2^ s^−1^ (mapped to an ionospheric reference altitude with a magnetic field intensity of 50,000 nT). The largest value of 2 × 10^9^ cm^−2^ s^−1^ corresponds to ~ 1.5 × 10^9^ cm^−2^ s^−1^ at 1000 km altitude [a magnetic field intensity of ~ 37,000 nT (Engwall et al. 2009)]. Even this extreme case is within the range covered by the dataset used in the present study.

## SZA dependence of the empirical relation between energy inputs and the ion number flux

### Empirical formula

Since the energy inputs and outflowing ion fluxes vary by multiple orders of magnitude, we investigated the relation in double logarithmic space according to the studies by Strangeway et al. (2005) and Brambles et al. (2011). Energy inputs (electron density in the loss cone, precipitating electron density proposed by Strangeway et al. (2005), DC and Alfvén Poynting fluxes) were logarithmically averaged using bins of the ion number flux (one order of magnitude was divided by 10 bins). The total latitudinal widths in ILAT of the outflow events were used as the weight for this averaging. The logarithmically averaged values (*F*_avg_) were fitted with a weighted least squares method in double logarithmic space using the following formula:

1 $$F_{i} = 10^{a} x^{b} \quad (\log_{10} F_{i} = a + b\;\log_{10} x)$$

where *F*_i_ is the ion number flux (mapped to 1000 km altitude) in cm^−2^ s^−1^, *x* is the energy input, and *a* and *b* are free parameters determined by the fitting. This fitting formula is the same as that used by Strangeway et al. (2005) and Brambles et al. (2011). In this fitting, the sum of the total latitudinal widths in ILAT of the outflow events (Δ_ILAT_) was used as the weight. We calculated *a* and *b* that minimize Σ((log_10_(*F*_avg_) – log_10_(*F*_i_(*x*)))^2^ Δ_ILAT_). The parameters selected as the energy input are those studied by Strangeway et al. (2005) and Brambles et al. (2011) and found that there are good correlations with outflowing ion fluxes. The use of other energy input parameters, to find which input parameter is good, and to investigate the functional shape are beyond scope of the present study.

As described above, we used logarithmically averaged energy inputs, not the outflow events themselves, for this fitting for the following reason, because the ion number fluxes used here are biased by the lower flux limit (10^7^ cm^−2^ s^−1^), which was used for event identification. Thus, in cases of small energy inputs, only cases in which the ion flux exceeded 10^7^ cm^−2^ s^−1^ were included for evaluation of the average energy input, despite that there must be cases where such a small energy input can cause ion outflows with the ion flux < 10^7^ cm^−2^ in reality. This limitation would uplift the small energy input part of the regression line, and makes the gradient of the line unrealistically gradual, if each of the outflow events were used for the fitting. Instead, the use of the averaged energy inputs for each level of the ion number flux helps us avoid such a bias, particularly for small energy input cases.

### Empirical relations between the electron density in the loss cone and the ion number flux

The electron density in the loss cone is defined as the partial electron density at the location of the satellite using 4 pitch angle bins around the precipitating direction (the center of pitch angle bins ranges from − 22.5° to 22.5° (Northern hemisphere) or from 157.5° to 202.5° (Southern hemisphere).

**Fig. 3:**
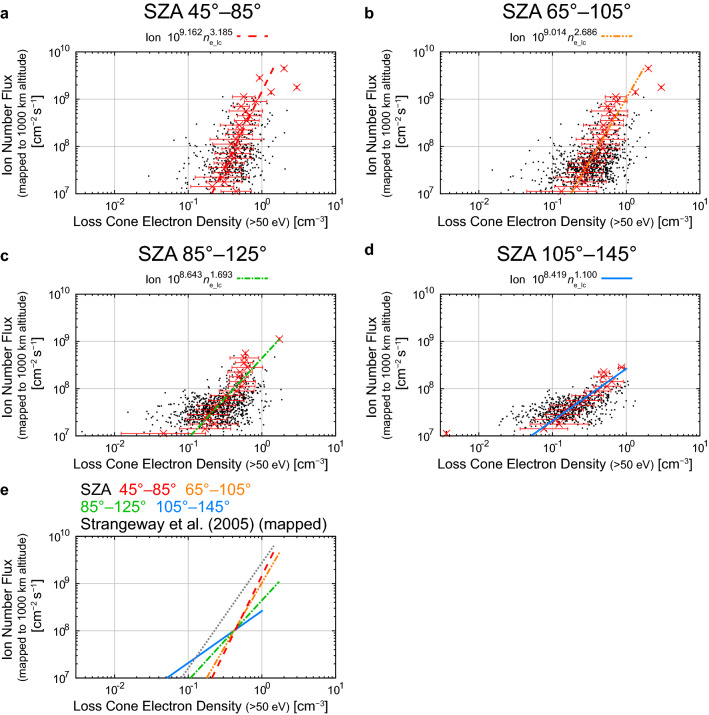
Relations between the electron density in the loss cone (< 50 eV) and the mapped outflowing ion number flux in the SZA ranges of **a** 45°–85°, **b** 65°–105°, **c** 85°–125°, and **d** 105°–145°, and **f** comparisons among the derived empirical relations in these SZA ranges and the empirical formula derived by Strangeway et al. (2005) (their Eq. 4) (multiplied by a factor of 2.9 to correct for the altitudinal difference of the ion number flux) (dotted gray line). Weighted averages and standard deviations of the logarithmic values are plotted as red crosses and solid lines (as error bars). Dotted lines indicate the empirical relations derived by the fitting. In Fig. 3e, the empirical relations in the SZA ranges of 45°–85°, 65°–105°, 85°–125°, and 105°–145° are shown using red-dashed, orange dashed-dotted-dotted, green dashed-dotted, and blue solid lines, respectively

Figure 3 shows the relations between the electron density in the loss cone (*n*_e_lc_) in cm^−3^ (> 50 eV) and the mapped ion number flux. The width of the SZA bins is 40°, and neighboring bins overlap in half (20°) to include a larger number of events in each SZA bin. This SZA range (45°–145°) includes 1563 out of 1569 events. The empirical formulas with 95% confidence intervals of the free parameters were derived as follows:

2 $${\text{SZA }}45^\circ {-}85^\circ :\;F_{i} = 10^{9.162 \pm 0.266} n_{{{\text{e}}\_{\text{lc}}}}^{3.185 \pm 0.708} ,$$

3 $${\text{SZA }}65^\circ {-}105^\circ :F_{i} = 10^{9.014 \pm 0.196} n_{{{\text{e}}\_{\text{lc}}}}^{2.686 \pm 0.431} ,$$

4 $${\text{SZA }}85^\circ {-}125^\circ :F_{i} = 10^{8.643 \pm 0.195} n_{{{\text{e}}\_{\text{lc}}}}^{1.693 \pm 0.340} ,$$

5 $${\text{SZA }}105^\circ {-}145^\circ :F_{i} = 10^{8.419 \pm 0.125} n_{{{\text{e}}\_{\text{lc}}}}^{1.100 \pm 0.172} .$$

The fitted line tends to become less steep with increasing SZA, although the difference in exponent between Eqs. 2 and 3 is well within the confidence intervals. The ion outflow events with small averaged number fluxes (~ 10^7^ cm^−2^ s^−1^) occur with smaller electron densities (~ 5 × 10^−2^ cm^−3^) at large SZA, while ion outflow events with large number fluxes (> 10^8^ cm^−2^ s^−1^) occur infrequently even in cases of high electron densities (> 5 × 10^−1^ cm^−3^). Above the electron density of ~ 4 × 10^−1^ cm^−3^ (the ion number flux of ~ 1 × 10^8^ cm^−2^ s^−1^), the ion outflow flux given by the empirical formulas (Eqs. 2–5) decreases with increasing SZA at a certain magnitude of the electron density (Fig. 3e). The exponents (*b*) of Eqs. 2 and 3 under sunlit conditions are slightly larger than those derived by Strangeway et al. (2005) (their Eq. 4, *b* = 2.240).

### Empirical relations between the precipitating electron density and the ion number flux

Strangeway et al. (2005) suggested the precipitating electron density (*n*_ep_), which has the dimensions of the number density in cm^−3^ defined as.

6 $$n_{{{\text{ep}}}} = 2.134 \times 10^{ - 14} f_{{{\text{en}}}}^{3/2} /f_{{{\text{ee}}}}^{1/2} ,$$

where *f*_en_ is the averaged field-aligned (downward positive) electron number flux (> 50 eV) in cm^−2^ s^−1^, and *f*_ee_ is the averaged field-aligned electron energy flux (> 50 eV) in mW m^−2^. Note that these fluxes are mapped to 1000 km altitude in the present study, while Strangeway et al. (2005) used local ones (~ 4000 km altitude). Thus, the precipitating electron density is ~ 2.9 times larger than that used by Strangeway et al. (2005) under the same condition. Positive values indicate downward fluxes. This precipitating electron density is presumably more useful for modeling studies than the electron density in the loss cone, since the precipitating electron density can be calculated using electron fluxes mapped along the field lines.

If the averaged energy flux and/or the averaged number flux were negative (upward), the precipitating electron density became negative (11 events) or imaginary numbers (40 events). Even after excluding such invalid cases, 1512 out of 1563 events (96.7%) remained available for this statistical analysis. (Same as “Empirical relations between the electron density in the loss cone and the ion number flux” section, 6 out of 1569 events were outside of this SZA range (45°–145°).) All excluded events except one have negative averaged number fluxes, which were significantly affected by upgoing low-energy electron beams (Ergun et al. 1998; Elphic et al. 2000; Andersson and Ergun 2006) in the region of ion outflow events. Most of these events occurred at large SZA (> 100°), which is consistent with the seasonal dependence of upward electron beams (Elphic et al. 2000).

**Fig. 4:**
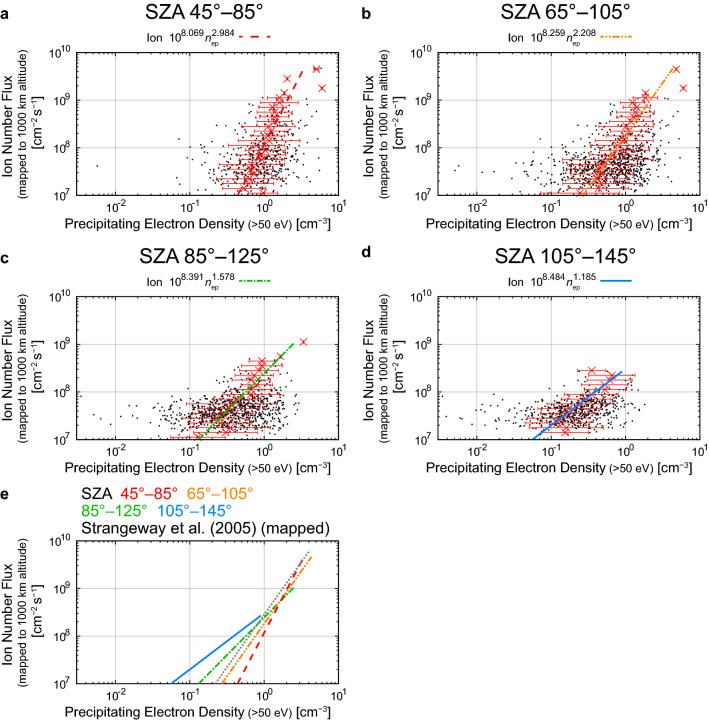
Relations between the precipitating electron density (< 50 eV) and the mapped outflowing ion number flux in the SZA ranges of **a** 45°–85°, **b** 65°–105°, **c** 85°–125°, and **d** 105°–145°, and **e** comparisons among the derived empirical relations in these SZA ranges and the empirical formula derived by Strangeway et al. (2005) (their Eq. 3) (multiplied by a factor of 2.9 to correct for the altitudinal difference of the ion number flux and the precipitating electron density) (dotted gray line). The format is identical to that of Fig. 3. There are 3 data points below precipitating electron density of 3 × 10^−3^ cm^−3^. The precipitating electron densities and the mapped outflowing ion number fluxes of the 3 events are 8.39 × 10^−5^, 6.31 × 10^−5^, and 1.91 × 10^−3^ cm^−3^, and 5.76 × 10^7^, 1.93 × 10^7^, and 4.85 × 10^7^ cm^−2^ s^−1^ at SZA of 94.7°, 99.8°, and 142.9°, respectively

The SZA dependence of the relations between the precipitating electron density and the ion number flux is shown in Fig. 4. The result is quite similar to that between the electron density in the loss cone and the ion number flux (Fig. 3), although the scatter of data points tends to be larger. The empirical formulas between the precipitating electron density (> 50 eV) and the mapped ion number flux are derived as listed below:

7 $${\text{SZA }}45^\circ {-}85^\circ :F_{i} = 10^{8.069 \pm 0.089} n_{{{\text{ep}}}}^{2.984 \pm 0.581} ,$$

8 $${\text{SZA }}65^\circ {-}105^\circ :F_{i} = 10^{8.259 \pm 0.088} n_{{{\text{ep}}}}^{2.208 \pm 0.342} ,$$

9 $${\text{SZA }}85^\circ {-}125^\circ :F_{i} = 10^{8.391 \pm 0.168} n_{{{\text{ep}}}}^{1.578 \pm 0.364} ,$$

10 $${\text{SZA }}105^\circ {-}145^\circ :F_{i} = 10^{8.484 \pm 0.178} n_{{{\text{ep}}}}^{1.1858 \pm 0.248} .$$

Below the precipitating electron density of ~ 1.5 cm^−3^ (the ion number flux of ~ 6 × 10^8^ cm^−2^ s^−1^), the ion outflow flux given by the empirical formulas (Eqs. 7–10), increases with increasing SZA at a certain magnitude of the precipitating electron density (Fig. 4e). The ion number flux given by these formulas tends to be slightly smaller than that derived by Strangeway et al. (2005) (their Eq. 3 after the altitudinal correction) under sunlit conditions.

### Empirical relations between the DC Poynting flux and the ion number flux

This SZA range (45°–145°) includes 1444 out of 1448 events. We excluded cases in which the averaged DC Poynting flux was negative (upward), and 1389 out of 1444 events (95.9%) remained available for this statistical analysis. Most of the excluded events (46 out of 59) occurred at large SZA (> 95°).

**Fig. 5:**
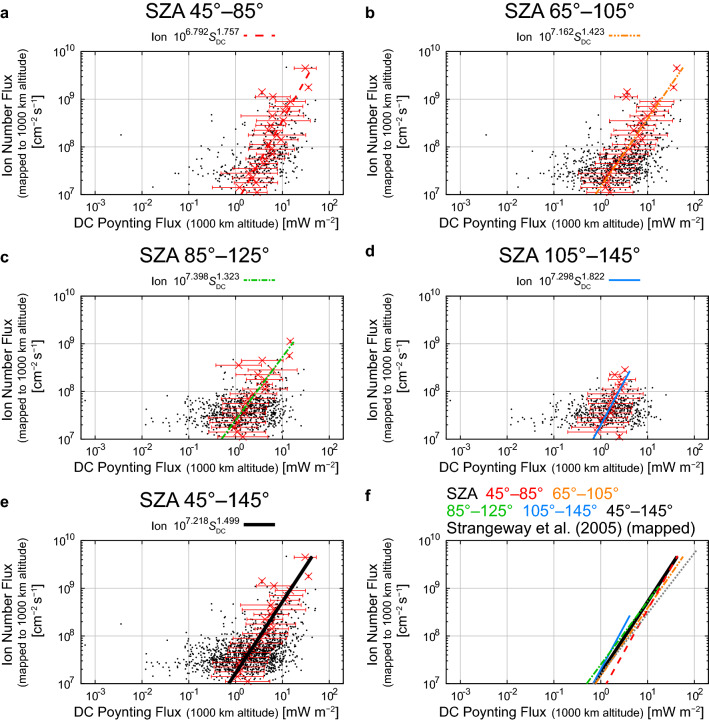
Relations between the mapped DC Poynting flux (< 0.125 Hz) and the mapped outflowing ion number flux in the SZA ranges of **a** 45°–85°, **b** 65°–105°, **c** 85°–125°, **d** 105°–145°, and **e** 45°–145° (all events), and **f** comparisons among the derived empirical relations in these SZA ranges and the empirical formula derived by Strangeway et al. (2005) (their Eq. 5) (multiplied by a factor of 2.9 to correct for the altitudinal difference of the ion number flux and the DC Poynting flux) (dotted gray line). The format of Fig. 5a–5e is identical to that of Fig. 3a–3d. In Fig. 5f, the empirical relations in the SZA ranges of 45°–145° (all data) are shown using a thick black line, in addition to the format of Fig. 3e

The relation between the DC Poynting flux and the ion number flux does not show clear SZA dependence, as seen from Fig. 5. The fitted functions are similar, but the large Poynting flux events tend to occur more under sunlit conditions than under dark conditions. Most of the averaged values of the DC Poynting flux in each flux bin at various SZA ranges are within the error bars (standard deviations) in the flux range where a significant number of events are present even at large SZAs. The empirical formulas between *S*_DC_ (mapped DC Poynting flux in mW m^−2^) and the mapped ion number flux are derived as listed below:

11 $${\text{SZA }}45^\circ {-}85^\circ :F_{i} = 10^{6.792 \pm 0.368} S_{{{\text{DC}}}}^{1.757 \pm 0.486} ,$$

12 $${\text{SZA }}65^\circ {-}105^\circ :F_{i} = 10^{7.162 \pm 0.119} S_{{{\text{DC}}}}^{1.423 \pm 0.209} ,$$

13 $${\text{SZA }}85^\circ {-}125^\circ :F_{i} = 10^{7.398 \pm 0.108} S_{{{\text{DC}}}}^{1.323 \pm 0.366} ,$$

14 $${\text{SZA }}105^\circ {-}145^\circ :F_{i} = 10^{7.298 \pm 0.114} S_{{{\text{DC}}}}^{1.822 \pm 0.500} .$$

Since the exponents did not show any systematic SZA dependence and indeed some of the derived exponents were within the 95% confidence intervals, we also calculated a regression line using all the events without classification of SZA:

15 $${\text{SZA }}45^\circ {-}145^\circ :F_{i} = 10^{7.218 \pm 0.084} S_{{{\text{DC}}}}^{1.499 \pm 0.181} .$$

The ion number fluxes from these formulas are roughly comparable to that from the empirical formula derived by Strangeway et al. (2005) (their Eq. 5 after the altitudinal correction) (Fig. 5f).

### Empirical relations between the Alfvén Poynting flux and the ion number flux

Same as “Empirical relations between the DC poynting flux and the ion number flux” section, 4 out of 1448 events were outside of this SZA range (45°–145°). After the exclusion of cases in which the averaged Alfvén Poynting flux was negative (upward), 1264 out of 1444 events (87.5%) remained available for this statistical analysis. Compared to the excluded cases of the DC Pointing Flux, the majority of excluded events are not observed under large SZAs (103 out of 180 at large SZAs (> 95°)).

**Fig. 6:**
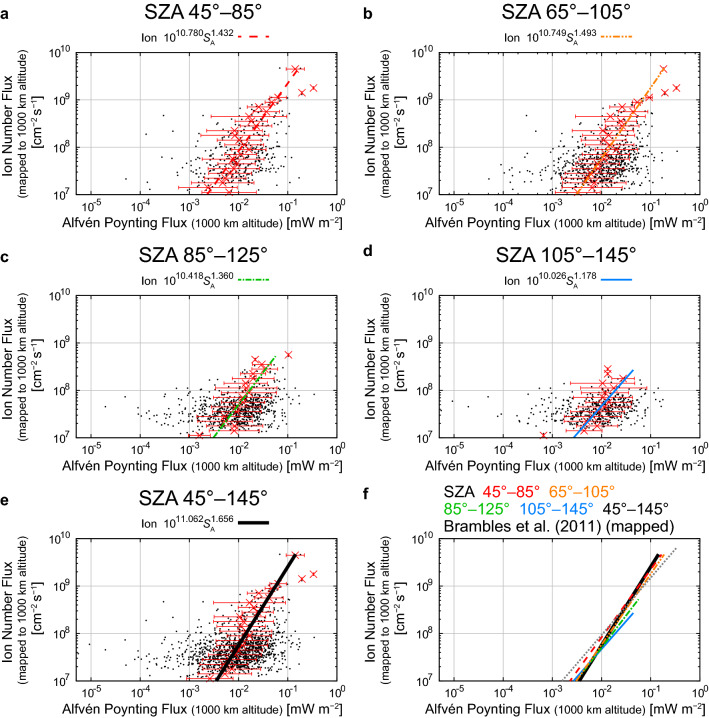
Relations between the mapped Alfvén Poynting flux (0.125–0.5 Hz) and the mapped outflowing ion number flux in the SZA ranges of **a** 45°–85°, **b** 65°–105°, **c** 85°–125°, **d** 105°–145°, and **e** 45°–145° (all events), and **f** comparisons among the derived empirical relations in the SZA ranges and the empirical formula derived by Brambles et al. (2011) (multiplied by a factor of 2.9 here to correct for the altitudinal difference of the ion number flux and the Alfvén Poynting flux) (dotted gray line). The format is identical to that of Fig. 5

Similar to the relations between the DC Poynting flux and the ion number flux (Fig. 5 and Eqs. 11–14), the relation between the Alfvén Poynting flux and the ion number flux does not show clear SZA dependence in the flux range where a large number of events are present, as seen from Fig. 6. The empirical formulas between *S*_A_ (mapped Alfvén Poynting flux in mW m^−2^) and the mapped ion number flux are derived as listed below:

16 $${\text{SZA }}45^\circ {-}85^\circ :F_{i} = 10^{10.780 \pm 0.698} S_{A}^{1.432 \pm 0.364} ,$$

17 $${\text{SZA }}65^\circ {-}105^\circ :F_{i} = 10^{10.749 \pm 0.630} S_{A}^{1.493 \pm 0.323} ,$$

18 $${\text{SZA }}85^\circ {-}125^\circ :F_{i} = 10^{10.418 \pm 0.726} S_{A}^{1.360 \pm 0.361} ,$$

19 $${\text{SZA }}105^\circ {-}145^\circ :F_{i} = 10^{10.026 \pm 0.851} S_{A}^{1.178 \pm 0.421} .$$

Since most of the derived exponents were within the 95% confidence intervals, we also calculated a regression line using all the events without classification of SZA:

20 $${\text{SZA }}45^\circ {-}145^\circ :F_{i} = 10^{11.062 \pm 0.675} S_{A}^{1.656 \pm 0.341} .$$

The ion number fluxes from these formulas are roughly comparable to that from the empirical formula derived by Brambles et al. (2011) (after the altitudinal correction)) (Fig. 6f).

## Discussion

The new empirical formulas derived in the present study include information about the SZA effect. This new information is valuable for investigating day–night and/or interhemispheric asymmetries (around solstice) of ion outflows in global magnetospheric models. Since the solar activity level (monthly mean *F*_10.7_ index) from January 1998 to February 1999 was almost the same as the latest solar maximum (Solar cycle 24), the empirical formulas obtained in the present study should be applicable to comparisons of the ion composition in the magnetosphere between the global models and measurements by the Van Allen Probes and the Magnetospheric Multiscale missions. Effects of solar activity will be studied in the future.

It is still impossible to determine the dominant energy input for the outflowing ion flux among the four on the basis of empirical formulas. One may think that the Poynting fluxes have a strong contribution, because the empirical formulas between the Poynting fluxes and ion number fluxes do not strongly depend on SZA. The error bars, which often spreads about an order of magnitude of the Poynting fluxes at a certain ion number flux (Figs. 5 and 6), however, tend to be larger than those of electron precipitation, which is usually within a factor of ~ 3–5 (Figs. 3 and 4). Since the low-energy ions have limited upward velocities (order of 10 km s^−1^), it takes at least several minutes for them to reach the altitude of 4000 km from the ionosphere. Thus, the energy inputs to the ionosphere at least several minutes before the satellite observations may be most relevant to the observed ion number fluxes at ~ 4000 km altitude. Observations by the Cluster spacecraft indicate that the O^+^ ion number flux fluctuates timescales of several minutes (Bouhram et al. 2004; Nilsson et al. 2008). This would imply that energy inputs that drive outflows also have fluctuations with similar timescales. Such fluctuations may explain the large error bars in Figs. 3, 4, 5, and 6. Nevertheless, the present results are based on a substantial number of events and we believe that the empirical relations can provide the average profile of ion outflow for varying energy inputs, which is readily usable for global magnetospheric simulations.

Even if the energy input is constant after a certain onset time, the outflowing O^+^ ion number flux increases dramatically in the initial ~ 10 min after the driving forces turned on in the models (Su et al. 1999; Horwitz and Zeng 2009). Whereas this time scale would change if different settings of the driving force are used, the duration of energy inputs would also contribute to the large deviation. If the intensity and duration of energy inputs are enough to modify and control the conditions of background plasma, SZA dependence would almost disappear (Horwitz and Zeng, 2009). That is, however, not the case for at least some events, because the empirical relation of the electron density in the loss cone or precipitating electron density and the outflowing ion number flux shows SZA dependence.

A combination of a latitudinally narrow cusp (Meng 1982, 1983; Kitamura et al. 2010a) and fast ionospheric convection during the main phase of geomagnetic storms causes ion energization with a short duration in a certain flux tube. In such cases, the duration of energization and the time-lag discussed above would be especially important (Varney et al. 2015) in addition to the energy input and SZA. Despite not being able to account for the time duration of the energy input, the derived empirical relations still provide average characteristics for the measured energy input with the error bars indicating variations partly due to different time durations and time-lags after effective acceleration.

Note that only ions above 10 eV are included in the present study. Since transverse energization of ions also occurs above ~ 4000 km altitude (Peterson et al. 1992; Miyake et al. 1993), the ion number flux above 10 eV for higher altitudes (for example, the inner boundary of magnetospheric simulations) is probably underestimated. During geomagnetic storms, O^+^ ion outflows with energies below ~ 10 eV with very large fluxes (> 10^9^ cm^−2^ s^−1^ mapped to 1000 km altitude) are present poleward of the cusp (observed at ~ 9000 km altitude) (Kitamura et al. 2010b). Such a population was not included in the present analysis owing to the difficulty in use of ion data below 10 eV, although how often such component becomes significant still remains as an open question, due to the lack of detailed ion observations below ~ 10 eV. This will become an important subject of observations in future missions.

As discussed in the introduction, empirical relations between energy inputs and ion outflow fluxes have been used as the boundary conditions of O^+^ ions at the inner boundary in global magnetospheric simulations (Fok et al. 2006, 2011; Moore et al. 2007, 2010; Brambles et al. 2010, 2011, 2013; Damiano et al. 2010; Peroomian et al. 2011; Ouellette et al. 2013). However, it is not clear whether O^+^ ions are dominant in many cases, because there are many observations of ion outflows with H^+^ ion fluxes larger than O^+^ ion fluxes (Tung et al. 2001; Peterson et al. 2001, 2006; Andersson et al. 2004; Wilson et al. 2004; Maes et al. 2015). The polar wind is present as thermal energy ion outflows (e.g., Yau et al. 2007 and references therein). Observational studies by Kitamura et al. (2016) showed that upward velocity of O^+^ ions are almost zero at least up to ~ 7000 km altitude in the sunlit polar cap region under geomagnetically quiet condition (the region and condition where very small auroral energy input are expected), while H^+^ ions have upward velocity at least above ~ 3000 km altitude. This fact clearly indicates that H^+^ ion outflows (polar wind type) do not need strong energy input, in contrast to O^+^ ion outflows. As for such H^+^ ions, different types of recent satellite observations (direct thermal energy ion measurements and estimations of components masked by spacecraft potential (Huddleston et al. 2005), measurements of spacecraft potential and wake (Engwall et al. 2009; André et al. 2015), and estimations using photoelectron outflows (Kitamura et al. 2012, 2015)) indicate that the number flux of the polar wind is ~ 2 × 10^8^ cm^−2^ s^−1^ (mapped to 1000 km altitude). This flux is larger than that for most of the events (especially for geomagnetically quiet periods), shown in Fig. 2. This polar wind type outflow is expected to exist also at the auroral zone. Thus, if background (polar wind) H^+^ ions can be accelerated up to 10 eV, additional O^+^ ions may not be necessary for driving ion outflows with small fluxes (< 10^8^ cm^−2^ s^−1^). Analyses that use mass resolved data (for example, the data from the Time-of-flight Energy, Angle, Mass Spectrograph (TEAMS) instrument on the FAST satellite (Klumpar et al. 2001), which are under re-calibration (Zhao et al. 2020)) will be important in the future, probably especially for ion outflow events with small fluxes.

## Summary and conclusions

To understand how strongly ionospheric conditions (sunlit or dark) affect ion outflows, we derived empirical formulas between energy inputs (electron density in the loss cone (> 50 eV), precipitating electron density (> 50 eV), mapped DC and Alfvén Poynting fluxes) and outflowing ion number fluxes (mapped to 1000 km altitude) for a wide range of SZA (45°–145°), using data obtained by the FAST satellite (3000–4150 km altitude) from 7 January 1998 to 5 February 1999 (monthly mean *F*_10.7_ index of 93.4–150.1).

Ion outflow events with large averaged fluxes (> 10^8^ cm^−2^ s^−1^) occur mostly under sunlit ionospheric conditions (SZA < 90°). Thus, the effect of the solar illumination (presumably high ionospheric density and/or large-scale height owing to high plasma temperature) probably prays an important role in the occurrence of ion outflows with large averaged fluxes.

Empirical relations between the electron density in the loss cone (> 50 eV) or precipitating electron density (> 50 eV) and the outflowing ion number fluxes show clear dependence on SZA at the ionospheric footprint. The outflowing ion number flux increases with increasing electron density in the loss cone and precipitating electron density, and the gradient of empirical formulas becomes less steep with increasing SZA. SZA dependence was not seen in the empirical relations between the Poynting fluxes (DC and Alfvén) and the outflowing ion number flux. Note that the electric fields perpendicular to the velocity vector of the satellite are not derived owing to the lack of reliable measurements of the electric fields along the spin axis. Thus, the magnitude of the Poynting fluxes is probably underestimated, and this incomplete Poynting flux measurement probably contributes to somewhat large scatter of the data points in the present analyses on the relationship between the Poynting fluxes and the ion flux.

Ionospheric conditions (sunlit or dark) affect ion outflows. Under dark ionospheric conditions, although weak electron precipitation can drive ion outflows with small averaged fluxes (~ 10^7^ cm^−2^ s^−1^), it is hard to drive intense ion outflows (> 10^8^ cm^−2^ s^−1^) presumably owing to low ionospheric O^+^ ion densities and/or a small scale height of O^+^ ions.

## Data Availability

The *Kp* index was provided by WDC for Geomagnetism, Kyoto. The EESA and IESA data and software for reading the data are available at http://sprg.ssl.berkeley.edu/data/fast/software/. The software for getting and reading the MGF and orbit data are available at http://sprg.ssl.berkeley.edu/fast/scienceops/fastidl.html. The monthly mean *F*_10.7_ solar radio flux index was provided by NGDC (http://www.ngdc.noaa.gov/stp/space-weather/solar-data/solar-features/solar-radio/noontime-flux/penticton/penticton_observed/listings/listing_drao_noontime-flux-observed_monthly.txt).

## Appendix A1. Calculation and subtraction of background of IESA

Background counts of IESA were subtracted from IESA data using count rates in the loss cone in the upward direction. The code used by Yao et al. (2008a, 2008b) was used for the calculation of the background count rates. It calculated the boundary of the loss cone as arcsin of square root of the ratio between the observed magnetic field intensity and the magnetic field intensity at the footprint (100 km altitude). If the center of the bin was > 5° inward of the boundary, the bin was regarded as inside the loss cone. Average energy fluxes inside the loss cone in the upward direction for each energy bin were calculated, and the minimum value for each time step was converted to the count rates. Although Yao et al. (2008a,b) used IESA data and EESA data to derive the background count rate, we only used IESA data to derive the background count rate, since the background count rates of IESA were slightly different from that of EESA. Another difference from the method of background subtraction by Yao et al. (2008a,b) is that the background count rate was calculated by a linear least-squares fitting using a moving window (25 s) for better handling of the data with various time resolutions, while they used boxcar averaged ones. This calculation was performed after the removals of spikes, which were presumably caused by erroneous data.

## Appendix A2. Rejection of erroneous data

### A2.1 Magnetic field data

In some cases, processed magnetic field data are apparently incorrect. To remove such incorrect data quantitatively as much as possible, the outflow events that satisfied the following two criteria at any of the 5-s averaged data points in the outflow regions were excluded from the present statistical analyses:

1. The magnetic field intensity that was calculated from observed data differs from that from the IGRF model by > 10%.
2. The direction of the magnetic field differs from that calculated using the IGRF model by > 5°.

Additionally, three events were excluded by visual inspection of the magnetic field data. Two of them (dayside part of orbit 6828 and nightside part of orbit 8271) included many spikes during the events and the remaining one (nightside part of orbit 6829) was on the way to gradually changing to incorrect values.

### A2.2 Electric field data

Sometimes an unusually large electric field was recorded just after a data gap. Thus, if there was any gap in the electric field data, the 5-s averaged Poynting flux at the period was not used. If Poynting fluxes were not available at any of data points in the outflow regions, the event was excluded from the statistical analyses in “Empirical relations between the DC poynting flux and the ion number flux” and “Empirical relations between the Alfvén poynting flux and the ion number flux” sections.

### A2.3 Ion and electron data

Sometimes ion or electron data are apparently incorrect. The ion data were excluded if counts at all pitch angle bins of IESA in one-third (top, middle, or bottom) of the energy bins were zero. This is the most typical type of the error. The counts do not become zero at such a large number of bins in the correct data (Fig. 1a and b). Frequently, another type of error occurs just after the change of the observational modes: Slow survey (~ 2.5 s resolution) and Fast survey (~ 0.625 or ~ 0.3125 s resolution). Seven (two) data points were excluded after the change to Fast (Slow) survey mode. This number was determined by visual inspection. The same rejection processes were also applied to electron data.

## Appendix A3. Periods of significant negative spacecraft charging

If the spacecraft is charged negatively, thermal energy ions are attracted from all directions. A 5-s period (one averaged data point) was regarded as a period of significant spacecraft charging, if there was at least one energy bin (4–70 eV) that the differential energy fluxes exceeded 5 × 10^6^ eV^−1^ cm^−2^ s^−1^ sr^−1^ eV^−1^ in all of the four pitch angle ranges: − 16.875°–16.875°, 40°–140°, 163.125°–196.875°, and 220°–320°. This threshold flux was determined by visual inspection of such events. The adjacent 5-s periods are also excluded for safety: some of data before averaging may be affected by the charging. By visual inspections of all outflow events, this definition is enough to exclude intervals of significant spacecraft charging with large ion number fluxes that can strongly affect the identification of outflow events. The upper energy limit of 70 eV is to avoid misidentification in the cusp in cases where ion precipitation was so intense that the differential energy flux exceeded 5 × 10^6^ eV^−1^ cm^−2^ s^−1^ sr^−1^ eV^−1^ even in the loss cone in the upward direction owing to pitch angle scattering.

## Appendix A4. Identification of the polar cap

The polar cap was defined with the use of 5-s averaged low-energy ion data (110 eV–24 keV), according to the threshold of a mean differential energy flux (< 10^4.6^ eV^−1^ cm^−2^ s^−1^ sr^−1^ eV^−1^) described by Andersson et al. (2004). The mean differential energy flux was calculated by using pitch angle ranges of − 30°–30°, 150°–210°, and 40°–140° or 220°–320°. In some orbits, contamination caused by solar radiation increases count rates around 90° or 270° at high latitudes. Because this increase affects the identification of the polar cap, the mean differential energy flux in the pitch angle range of 40°–140° or 220°–320°, whichever smaller, is selected to avoid this contamination (Kitamura et al. 2015). Continuous (≥ 10 s, ≥ 2 data points) periods in which the mean differential energy flux met the criterion were selected as candidates of the polar cap. Sometimes this criterion was satisfied for data obtained in the subauroral zone. To exclude such cases, the candidates that are connected to the region where energetic ions (> 4 keV) show double loss cones (Appendix A5) without a data gap of ≥ 60 s or equatorward of such regions were excluded. In some cases, short candidates that appeared between the auroral zone and the region of the double loss cone could not be excluded. There were some cases in which the region of double loss cone was not identified and candidates in the subauroral zone could not be excluded. All these two types of cases, however, had polar cap periods much longer than 200 s, and thus the overlooking did not affect the exclusion of the outflow events.

Although contamination owing to solar radiation causes increase in count rates, the increase occurs around the pitch angle of 90° at high latitudes. Thus, this does not strongly affect the calculations of field-aligned ion fluxes in the outflow regions (> 10^7^ cm^−2^ s^−1^). This is one of the reasons why we set the lower flux limit to identify the outflow regions. In some cases, the contamination causes the apparent field-aligned ion fluxes of the order of 10^6^ cm^−2^ s^−1^ (mapped to 1000 km altitude). To treat ion outflows with fluxes smaller than ~ 10^7^ cm^−2^ s^−1^ in the future, this apparent flux must be corrected.

## Appendix A5. Identification of double loss cones and the subauroral zone

Identification of regions of double loss cones was performed if the mean differential energy flux of ions above 4 keV in the pitch angle ranges of 40°–140° and 220°–320° (trapped population) were larger than 10^4.6^ eV^−1^ cm^−2^ s^−1^ sr^−1^ eV^−1^. The periods of double loss cones were defined as cases where the mean differential energy flux above 4 keV near the center of the loss cone (in the pitch angle range from 163.125° to 196.875° (Northern Hemisphere) or from − 16.875° to 16.875° (Southern Hemisphere)) was lower than 50% of those in the pitch angle ranges of 40°–140° and 220°–320°. Examples are shown above Fig. 1 with red bars. Even if there were data gaps in the interval of double loss cones, the interval was treated as one continuous interval (~ 1940 UT). To avoid misidentifications, short intervals (1 or 2 data points with double loss cones) were excluded.

Very energetic ion conics that extended above 4 keV could be misidentified as a region of double loss cones, although such cases were very rare at this altitude. Thus, in the case in which the ion number flux above 4 keV exceeded 10^6^ cm^−2^ s^−1^ (mapped to 1000 km altitude), the region was treated as the region of double loss cones only if both sides of the case satisfied the criteria of double loss cones.

The region of ILAT < 45° or high background count rates (> 50 counts/s) that were connected to ILAT < 65.9° (*L* < 6) were removed (marked as subauroral zone). In this removal, even if there were data gaps in the interval of high background count rates, the interval was regarded as connected. To focus on outflows in the auroral zone, the poleward boundary of the most equatorward region of double loss cones in the remaining part of the inbound or outbound pass was selected as the equatorward boundary of the region for the analyses (poleward of the subauroral zone). Examples of the identified subauroral zones are shown above Fig. 1 with black bars.

## Abbreviations

EESA: Electron spectrometers
FAST: Fast Auroral Snap shoT
IESA: Ion spectrometers
IGRF: International Geomagnetic Reference Field
ILAT: Invariant latitude
MLT: Magnetic local time
SZA: Solar zenith angle
